# Implementation of multiple-domain covering computerized decision support systems in primary care: a focus group study on perceived barriers

**DOI:** 10.1186/s12911-015-0205-z

**Published:** 2015-10-12

**Authors:** Marjolein Lugtenberg, Jan-Willem Weenink, Trudy van der Weijden, Gert P. Westert, Rudolf B. Kool

**Affiliations:** Scientific Institute for Quality of Healthcare (IQ healthcare), Radboud university medical center, P.O. Box 9101, 6500 HB Nijmegen, The Netherlands; Scientific center for care and welfare (Tranzo), Tilburg School of Social and Behavioral Sciences, Tilburg University, P.O. Box 90153, 5000 LE Tilburg, The Netherlands; School for Public Health and Primary Care (CAPHRI), Department of General Practice, Maastricht University, P.O. Box 616, 6200 MD Maastricht, The Netherlands

**Keywords:** Clinical decision support, Clinical practice guidelines, Primary care, Barriers, Interventions, Implementation

## Abstract

**Background:**

Despite the widespread availability of computerized decision support systems (CDSSs) in various healthcare settings, evidence on their uptake and effectiveness is still limited. Most barrier studies focus on CDSSs that are aimed at a limited number of decision points within selected small-scale academic settings. The aim of this study was to identify the perceived barriers to using large-scale implemented CDSSs covering multiple disease areas in primary care.

**Methods:**

Three focus group sessions were conducted in which 24 primary care practitioners (PCPs) participated (general practitioners, general practitioners in training and practice nurses), varying from 7 to 9 per session. In each focus group, barriers to using CDSSs were discussed using a semi-structured literature-based topic list. Focus group discussions were audio-taped and transcribed verbatim. Two researchers independently performed thematic content analysis using the software program Atlas.ti 7.0.

**Results:**

Three groups of barriers emerged, related to 1) the users’ knowledge of the system, 2) the users’ evaluation of features of the system (source and content, format/lay out, and functionality), and 3) the interaction of the system with external factors (patient-related and environmental factors). Commonly perceived barriers were insufficient knowledge of the CDSS, irrelevant alerts, too high intensity of alerts, a lack of flexibility and learning capacity of the CDSS, a negative effect on patient communication, and the additional time and work it requires to use the CDSS.

**Conclusions:**

Multiple types of barriers may hinder the use of large-scale implemented CDSSs covering multiple disease areas in primary care. Lack of knowledge of the system is an important barrier, emphasizing the importance of a proper introduction of the system to the target group. Furthermore, barriers related to a lack of integration into daily practice seem to be of primary concern, suggesting that increasing the system’s flexibility and learning capacity in order to be able to adapt the decision support to meet the varying needs of different users should be the main target of CDSS interventions.

## Background

Over the past years there has been an increase in the availability of computerized decision support systems (CDSSs) in all areas of healthcare, including the primary care setting. CDSSs are information systems designed to optimize clinical decision making [1]. By matching characteristics of individual patients to a computerized medical knowledge base CDSSs can provide patient-specific recommendations to healthcare providers during patient consultations. In this way, they have the potential to improve quality of care [2, 3].

Despite the increased availability of CDSSs in various healthcare settings, the use of these systems in practice is still limited [4]. Not surprisingly, conclusive evidence on their effectiveness in improving quality of care also remains to be established [5]. Whereas some reviews have shown that CDSSs can improve medical practice, they do not always result in improvements [1, 6–10]. Moreover, the effects of CDSSs on patient outcomes have been less studied and results have been less favorable and less consistent [10–16].

To improve the use and effectiveness of CDSSs, insight is needed into users’ perceived barriers to using CDSSs in practice. Several studies have been conducted to identify the factors that physicians perceive as hindering implementation [4, 12, 17–21]. These studies indicate that patient-related factors (e.g. effects on patient communication) and environmental factors (e.g. organizational context) are considered as important barriers to implementing CDSSs in practice. The body of evidence on barriers and how to overcome them, however, is still limited [4].

Most barrier studies have focused on CDSSs that are aimed at a limited number of decision points e.g. [12, 22] rather than on multiple-domain covering CDSSs targeting multiple groups of users. In addition, these CDSSs have usually been tested in selected small-scale academic settings rather than being practice-driven and implemented at a large scale. Large scale implemented CDSSs in primary care in which several types of primary care practitioners (PCPs) work with a multiple-domain covering CDSS [23] may yield different types of barriers among their users.

The aim of this study was therefore to identify the perceived barriers to using large-scale implemented CDSSs, covering multiple disease areas in primary care. In addition, interventions to improve the use of CDSSs as suggested by the target group were identified. We included all types of PCPs that could potentially work with CDSSs in our study, rather than just general practitioners (GPs), as to maximize the generalizability of our findings. This paper focuses on the perceived barriers; results on suggested interventions will be described elsewhere.

## Methods

### Setting

In the Netherlands, there are approximately 5,000 general practices. Within these practices nearly 11,000 GPs are delivering care [24]. More than 1,700 medical doctors are in training to become GPs [24]. These GP trainees all have completed a 6-year master program in general medicine and work 4 days a week during an average period of three years in a group practice under supervision of an experienced GP. One day per week is focused on educational activities and group meetings in which daily problems are discussed and videotapes are sometimes presented [25]. Aside from GPs and GP trainees, between 3.700 and 4.700 practice nurses (PNs) work within 75 % of these general practices [26]. They are mainly responsible for providing basic care such as regular check-ups for patients with a chronic illness and completing their patient files. Together, these PCPs (GPs, GP trainees and PNs) are responsible for providing primary care in Dutch general practices. Currently, a total of seven major different electronic health record systems (EHRS) are used.

### CDSS initiative in Dutch general practice: NHGDoc

Within the Dutch primary care setting there is one main CDSS initiative, which is called NHGDoc. NHGDoc is a CDSS initiated and developed in 2006 by ExpertDoc BV and currently owned by the Dutch College of General Practitioners (DCGP, NHG in Dutch). NHGDoc is integrated by web services within the electronic health record system (EHRS) and is based on the NHG guidelines, the prevailing guidelines for general practice in the Netherlands [27]. It provides GPs, GP trainees and PNs evidence-based and, on the basis of structured data in the EHRS, patient-specific advices during consultation in terms of patient data registration, drug prescription and management [28].

At the time of conducting the focus group study NHGDoc covered the following disease areas: Cardiovascular risk management, Asthma/COPD, Diabetes mellitus type II, Thyroid disorders, Viral hepatitis and other liver diseases, Atrial fibrillation and Subfertility, Gastro protection and Chronic renal failure. For each NHG guideline key recommendations have been selected based on relevance of disease burden, revision status of the guideline, and opportunity to translate or normalise the recommendation into if-then rules. This selection of key recommendations is approved by representative experts of the guideline committees. Subsequently, the selected key recommendations are digitized, thoroughly tested and deployed into the NHGDoc system. The total number of key recommendations/advices that could be shown per domain varies from 50–250 key recommendations/advices. However, the average number of key recommendations/ advices that is shown per patient encounter is 7.2, based on an average number of 2.3 domains/disease areas.

At the time of conducting this study, NHGDoc was integrated in 6 out of 7 major EHRSs being used in Dutch general practice, covering approximately two-third of all Dutch general practices. NHGDoc had been available for approx. 2.5 years for MicroHIS X users, 2 years for Promedico-ASP users and less than half a year for users of the other included EHRSs.

### NHGDoc - Basic functions: alerts and feedback

**Fig. 1:**
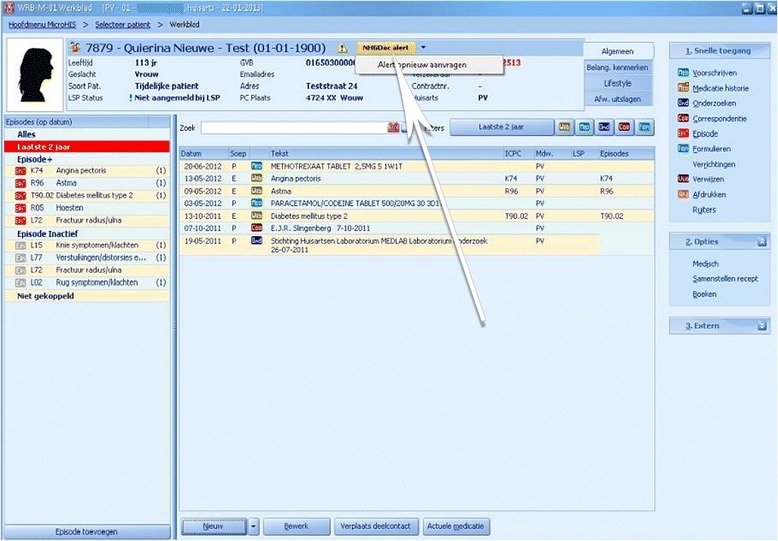
Appearance of an NHGDoc alert button in an EHRS (MicroHIS X). Copyright: iSOFT NEDERLAND B.V., Mendelweg 32, 2333 CS Leiden, the Netherlands

When a PCP opens a patient file in the EHRS, anonymous patient data and medical performance data are sent to the NHGDoc server. The data are then compared to the digitized guideline recommendations and in case of a discrepancy between current and advised care, an alert will be sent back to the PCP. By default, the NHGDoc alert button is displayed in green, but turns into yellow when a discrepancy is detected (see Fig. 1). It is up to the PCP to open the NHGDoc alert or not. In the Promedico EHRS, the alert button could also be gray, in which case the user would need to manually request the alert.

**Fig. 2:**
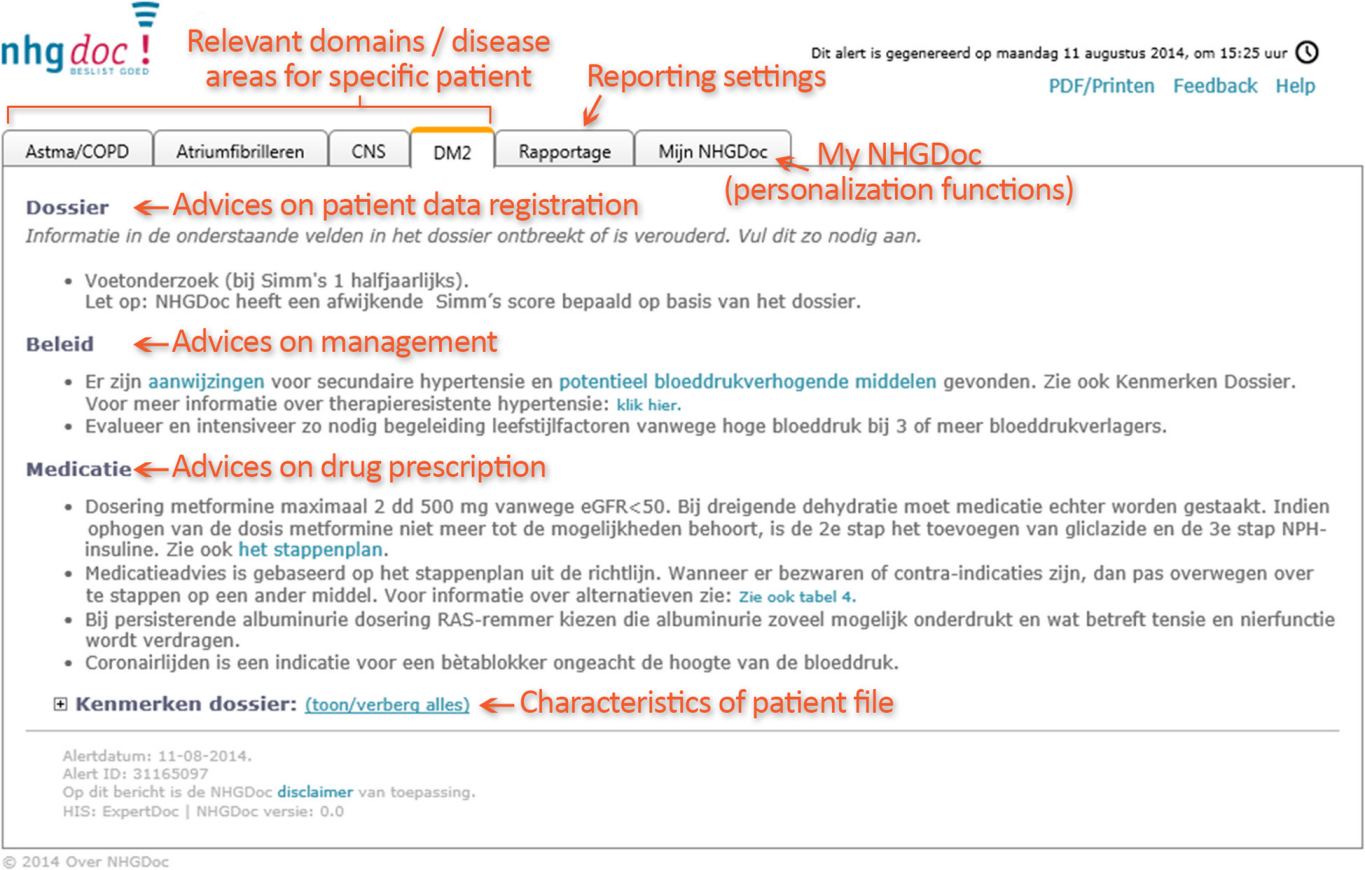
Example of an NHGDoc alert. Copyright: ExpertDoc B.V., Veerkade 8d, 3016 DE Rotterdam, the Netherlands

When the GP (trainee) or PN clicks on the yellow NHGDoc alert button, an alert window appears (see Fig. 2). For each relevant domain, the NHGDoc alert includes up to three types of patient-specific advices: on patient data registration, on management and on drug prescription. The alert is sensitive to the specific patient case (based on patient-specific ICPC (International Classification of Primary Care) codes, NHG Lab codes (codes used by the Dutch College of General Practitioners for laboratory and other diagnostic tests and results) and ATC (Anatomical Therapeutic Chemical Classification System) codes), and generates feedback by showing the recommendation(s) for which discrepancies were found as compared to the guideline recommendations. At the bottom of the alert, the patient profile (characteristics of patient file) consisting of the data on which the advices are based, is shown.

By clicking on the Feedback link at the top right corner, users have the option to ask or provide feedback to/from ExpertDoc (the organization that has developed and maintains NHGDoc) about the received alert. ExpertDoc also informs the NHG about the received user feedback.

### NHGDoc - Personalization functions: Tailoring My NHGDoc to PCPs’ specific needs

Aside from the basic functions, NHGDoc allows the user to adapt the decision support to meet their personal preferences in two different ways.

#### Alert settings

Users can adjust the preferences of the alerts to match their personal needs. They can choose to switch alerts on and off on demand at several levels: the system (NHGDoc as a whole – only in the EHRS MicroHIS X), the modules (NHGDoc domains), the types of alerts (patient data registration, management, drug prescription), and the patients. NHGDoc can also be used as an educational tool for specific domains, for example, a practice can choose a temporary switch-on of a module to generate input for small-group peer review and quality improvement activities.

#### Reporting settings

Users also have the option to request specific reports with respect to the number and types of alerts they have received per domain within a specific period of time (per year, per month, per week or per day).

### Study design

We used a qualitative study design including three 1.5-h focus groups. Focus groups have proven to be useful as a method of providing in-depth information and for exploring cognitions and motivations underlying behavior [29–32]. The focus group sessions therefore enabled us to identify the perceived barriers to using CDSSs in primary care among the target groups of users.

The focus group study, of which the results are presented in this paper, is part of a larger evaluation study on the effectiveness of NHGDoc in improving quality of primary care [33]. The need for ethical approval for the NHGDoc evaluation study was waived by the research ethics committee of the Radboud university medical center.

This focus group study has been designed and reported (whenever applicable) in accordance with the RATS guidelines [34].

### Selection of participants

To select participants we sent a direct email to all 233 practices that participated in the NHGDoc evaluation study [33]. All PCPs working within these practices (GPs, GP trainees and PNs) were invited to participate in the focus group study. After two weeks a first reminder was sent and after four weeks we sent a second reminder. Also, an invitation was sent by email to all medical doctors receiving training as a GP at the University Medical Centre in Utrecht, the medical centre located in the area in which the focus groups were to be conducted. Additional announcements were placed on relevant websites, in newsletters and through social media (i.e. Facebook, Twitter and LinkedIn). PCPs could register for one of the three focus groups organized. All participants received a gift voucher of €100, − and were offered reimbursement of their travel expenses.

### Focus group sessions

The focus group sessions were conducted at the NHG, which is located in the center of the Netherlands. In each focus group session, the PCPs had a semi-structured discussion about their perceived barriers to using CDSSs such as NHGDoc in primary care. The sessions were moderated by ML and RBK (principal investigators of the NHGDoc evaluation study and experienced moderators of focus groups) and by a representative of the NHG.

Prior to the formal start of the discussion, one of the moderators gave instructions about the focus group session and explained that the responses of the participants will remain anonymous and that their names will not be mentioned in publications. In addition, the moderators’ independence towards NHGDoc, the main CDSS in Dutch general practice, was emphasized. Representatives of ExpertDoc, the organization that has commercial interests in NHGDoc, were deliberately not invited to the focus group sessions. Participants were asked for their approval to participate and gave permission to audio-tape the session.

A predefined topic list was used to structure the discussion. This list consisted of the following broad themes: the value of CDSSs in a primary care setting, CDSSs in an ideal world, experiences with using CDSSs with the example of NHGDoc, perceived advantages and disadvantages, and barriers to using them in practice. The three focus group sessions were audio-taped.

### Data analysis and synthesis

The focus group sessions were transcribed verbatim. Two researchers (ML and JWW) independently studied the transcripts in Atlas.ti 7.0. They first independently created a code list consisting of the main barriers to using CDSSs. After studying the first half of the first transcript, the two code lists were compared and discrepancies were discussed until consensus was reached. This process was repeated after studying the second half of the first transcript. The remaining transcripts were categorized using the mutually agreed on code list.

Next, the code list was discussed and emerging themes were grouped by theory-based categories. As a basis we used the framework of Cabana [35], which presents barriers to using clinical practice guidelines, complemented with literature focusing on barriers to using CDSSs [4, 17, 18]. The information in each category of barriers was reflected on and interpreted jointly. This process resulted in the framework of barriers to using CDSSs presented in Table 2.

## Results

### Description of participants

Twenty-five PCPs registered for one of the three focus groups of which 24 participated (96 %), varying from 7 to 9 per session. Fifty-four percent of the participants were male (*n* = 13); their mean age was 47 years (see Table 1). Most of the participants worked as a GP (*N* = 15); followed by PNs (*N* = 5) and GP trainees (*N* = 4). The majority of the participants worked in a duo practice (*n* = 9) and used the EHRS MicroHIS X (*n* = 12).

From the group of GPs (*N* = 15), the majority was male (80 %), were aged 45 years or older (71 %) (mean age = 52) and worked in either a solo practice or a duo practice (both 43 %). Compared to the total population of Dutch GPs [24], GPs in our sample were more often male (80 % versus 57 %), were somewhat older (52 versus 48.5 years) and worked relatively less often in group practices (14.2 % versus 36 %). The GP trainees were predominantly female (75 %) and had a mean age of 30 years. From the group of PNs all (*n* = 5) were female; their mean age was 47 years.

**Table 1:** Characteristics of participants

	Number	Percent	Mean
All PCPs	24		
Sex			
Male	13	54	
Female	11	46	
Age *(in years)* (*n* = 23)			47
Type of practice (*n* = 21)			
Solo	8	38	
Duo	9	43	
Group (>2)	4	19	
Type of EHRS (*n* = 22)			
MicroHIS X	12	55	
Promedico-ASP	7	32	
Other EHRs	3	14	
GPs	15		
Sex			
Male	12	80	
Female	3	20	
Age *(in years)* (*n* = 14)			52
GP trainees	4		
Sex			
Male	1	25	
Female	3	75	
Age *(in years)*			30
Practice nurses	5		
Sex			
Male	0	0	
Female	5	100	
Age *(in years)*			47

*PCPs* primary care practitioners, *EHRS* electronic health record system, *GPs* general practitioners, *GP trainees* general practitioners in training

### Perceived barriers

Three main types of barriers emerged: knowledge-related barriers, barriers related to the evaluation of the features of the CDSS (source and content, format/lay out, and functionality), and external barriers interacting with the CDSS (patient-related and environmental factors) (see Table 2).

**Table 2:** Framework of barriers to using CDSSs

Knowledge-related barriers
- 1. Knowledge regarding the (specific functions of the) CDSS
o Knowledge of basic functions
o Knowledge of user personalization functions
Barriers related to the evaluation of the features of the CDSS
- 2. Source and content of the CDSS
o Reliability of the source of the content
o Currentness of the content
o Relevance of the alert content for different user groups
o Relevance of the alert content for individual users, with varying needs across time
- 3. Format/lay out of the CDSS content
o Notification method of alerts (too intrusive or uninformative)
o Readability of the alert text (too wordy/verbose)
- 4. Functionality of the CDSS
o Responsiveness of the system (loading of an alert takes too long)
o Intensity of alerts (low threshold for triggering alerts)
o Flexibility (lack of adjustability to personal preferences)
o Learning capacity of the system (only fixed rules are used)
External barriers interacting with the CDSS
- 5. Patient-related factors
o Doctor-patient communication (too much time spent on the computer during consultation)
o Relevance of alert content for patient (discrepancy between patient’s reason for visit and alert content)
- 6. Environmental factors
o Limited time available (during and after consultation)
o Too much additional work required (during and after consultation)
o Lack of integration with other systems (no direct links to follow-up actions)
o Fear for misuse of data (patient data and medical practice) by third parties (i.e. health insurers)

*CDSS* computerized decision support system

#### Knowledge-related barriers

##### Knowledge regarding the (specific functions of the) CDSS

Lack of knowledge regarding how the CDSS works appeared to be a barrier to using it. Both insufficient knowledge regarding the basic functions of the CDSS (i.e. alerts and feedback) as well as regarding options to adapt the decision support to personal preferences were mentioned as barriers among the PCPs (see Table 3). Also, most of the PCPs reported never to have received a formal introduction of the system and/or education or training on how to use the CDSS.

**Table 3:** Examples of perceived barriers related to knowledge regarding the (specific functions of the) CDSS

- Lack of knowledge regarding basic functions
• “I have no idea what this grey button [manually to be requested alerts] means. It used to have a color and now it’s grey so I think something is wrong”.
• “I didn’t even know there was a feedback option, never heard of it before”.
- Lack of knowledge regarding personalization functions
• “I had no idea about all these options! Now, I’m a lot more enthusiastic. I’m gonna use it right away!”.

#### Barriers related to the evaluation of the features of the CDSS

##### Source and content of the CDSS

PCPs mentioned that the reliability of the source of the content (the initiator of the alerts) was a barrier to using it. Some users mentioned that they questioned whether the pharmaceutical industry was involved in determining the therapeutic recommendations (see Table 4). The content of the decision support was also perceived as a barrier among the PCPs: they sometimes doubted the currentness and therefore the reliability of the content as they believed that it might take some time before a revised guideline is updated in the system. Also, the PCPs agreed that the alert content was not always consistent with the varying needs of different user groups (GPs, GP trainees and PNs), nor with the varying needs of individual users across time (see Table 4).

**Table 4:** Examples of perceived barriers related to the source and content of the CDSS

- Lack of trust in reliability of the source of the content
• “Well, then it makes me wonder: do they own any stock options? Yeah, I know it sounds a bit silly. But it makes me wonder which pharmaceutical company is backing this?”.
- Lack of trust in currentness of content
• “How current are the guideline recommendations? Are the alerts really up to date? That’s what you [the researchers] should include in your advice, that the content of NHGDoc should be updated on a daily basis”.
- Irrelevant alerts for different user groups
• “It shouldn’t be necessary to override so many alerts; only the sections that apply to us [PNs] should be highlighted”.
- Irrelevant alerts for individual users, with varying needs across time
• “Well, for example, you don’t wanna see the ‘advice to give up smoking alert’ again, when it’s already clear that it aint gonna happen with this patient. You don’t want to receive that alert over and over again”.

*PNs* practice nurses

##### Format/lay out of the CDSS content

The format or lay out of the content of the CDSS was also mentioned as a barrier (see Table 5). With respect to the notification method of the alert both too intrusive alerts (e.g. pop-ups in the middle of the screen) as well as uninformative alerts (e.g. just a small green button with the text alert on it) were considered as barriers. PCPs also mentioned the readability of the alert text, which they often considered too verbose.

**Table 5:** Examples of perceived barriers related to the format/layout of the CDSS content

- Notification method (too intrusive or uninformative)
• “A pop-up means an additional action which might not be convenient at that time. Now, it’s under my own control”.
• “So, you should immediately see whether it concerns a content alert or an alert regarding patient data registration. And also the subject: diabetes, cardiovascular risk management….If you move your mouse over the alert you should be able to see it. That would be worth a whole lot!”.
- Readability of the alert text (too wordy/verbose)
• “I think the phrasing is sometimes very complex. ‘Research has shown that….’ or ‘You could consider…..’. This should be a bit more to the point really!”.

##### Functionality of the CDSS

The participants indicated that the functionality of the CDSS was also perceived as a barrier to using it (see Table 6). First of all, the responsiveness of the system was mentioned to be a problem with the loading of alerts sometimes taking too long. Also, the intensity of alerts (low threshold for triggering alerts) was mentioned to be a barrier. Participants felt that the frequency of alerts, particularly with respect to patient data registration, was too high. In addition, a perceived lack of flexibility of the system in terms of being able to adapt the content of the decision support to personal preferences was perceived as a barrier to using a CDSS. Also, a lack of learning capacity of the system was indicated to be a barrier with the system using only fixed rules rather than learning from the PCPs' use of the system and adjusting the content accordingly.

**Table 6:** Examples of perceived barriers related to the functionality of the CDSS

- Responsiveness of the system (loading of an alert takes too long)
• “I gave up rather quickly because the loading of an alert took way too long”.
- Intensity of alerts (low threshold for triggering)
• “So it shouldn't be too much, not like ten alerts per patient right? Then you’ll get a little over-alerted right? Enough is as good as a feast!”.
• “… did you check kidney function, liver function…? At a certain point you’ll get overloaded with information that is actually quite straightforward…. 25 yellow *[an alert]* out of the last 50 patients....”.
- Lack of adjustability to personal preferences
• “The customization options are still rather limited. You should be able to turn off specific types of advices, for instance the ‘give up-smoking-alerts’ rather than all life style advices at ones”.
• “I wanna be able to set the threshold myself, so not all at 40 for blood pressure, for example”.
- Lack of learning capacity of the system
• “This almost asks for a system that can be overruled. You don’t want the computer stupidly, not intuitively, to state the same thing over and over again. In practice, that will result in overriding alerts. The system should cooperate with how people think”.

#### External barriers interacting with the CDSS

##### Patient-related factors

Patient-related factors were also perceived as barriers to using CDSSs. Many PCPs mentioned that using CDSSs has a negative effect on patient communication during consultation and is considered as a barrier to using them. Also, the discrepancy between the patient’s reason for visiting the PCP and the content of the alert was a reason not to use it (see Table 7).

**Table 7:** Examples of perceived barriers related to patient factors

- Doctor-patient communication (too much time spent on the computer during consultation)
• “It just takes a lot of time and makes you focus too much on your computer and the patient just does not like that. I can see the patient thinking… while I’m only staring at that stupid screen”.
• “I click [on the computer] like there’s no tomorrow, also during patient consultation with the patient next to me. And I sometimes find it disturbing, that I spend so much time on the computer…”.
- Relevance of alert content for patient (discrepancy between patient’s reason for visit and alert content)
• “The patient’s reason for visiting that absolutely does not match the content of the alert. If someone visits with his ankle, you don’t want to receive an advice on statins”.

##### Environmental factors

Environmental factors were also commonly reported as a barrier to using CDSSs. PCPs mentioned the limited time during consultation, which makes it difficult to use the CDSS, as well as the additional work it requires to use the CDSS (see Table 8). In addition, the PCPs reported that CDSSs were often not linked to other systems with follow-up actions such as the electronic prescribing system or test ordering forms, which makes it complex to use them. Moreover, some PCPs indicated that they had concerns regarding the misuse of data (patient data and medical practice) by third parties (i.e. health inspectorate or health insurance companies).

**Table 8:** Examples of perceived barriers related to environmental factors

- Limited time available
• “In daily practice I can’t manage to create time for this. It just doesn’t fit in the regular consultation hours”.
- Too much additional work required
• “These systems, the way they’re currently introduced, just take too much time. And then I deliberately choose not to use them”.
• “It’s a lot of extra work! It has almost become a task of its own. With all the items you have to fill out”.
- Lack of integration with other systems
• “The alert screen should directly be linked to follow-up actions that need to be done! So, if you are to prescribe a statin, it should go directly to that screen. If you have to register blood pressure, you should be able to register it right there”.
- Fear of misuse of data by third parties
• “And then the healthcare inspectorate comes down to visit and asks: why didn’t you do this or that when there was an alert. It shouldn’t be used for this purpose!”.

## Discussion

In this focus group study we identified the barriers to using large-scale implemented CDSSs covering multiple domains of care in general practice as perceived by Dutch PCPs. We found that three groups of barriers may hinder implementation: knowledge-related barriers, barriers related to the evaluation of the features of the CDSS and external barriers interacting with the CDSS. Particularly, insufficient knowledge regarding the CDSS and barriers related to a lack of integration into daily practice such as too high intensity of alerts, irrelevant alerts, and the additional time it requires to use the CDSS were commonly perceived barriers. Aside from a proper introduction of the system to the target group, our results indicate that improving the flexibility and learning capacity of the systems and thereby increasing options to adapt the decision support to meet the varying needs of different users, should be the target of improved CDSS interventions.

Prior studies on barriers to using CDSSs found that particularly external factors (i.e. patient-related factors and environmental factors) were perceived as barriers among users [4, 17–21] Consistent with these studies we found that external barriers were indeed relevant. However, we also identified commonly perceived knowledge-related barriers. These may particularly apply to large-scale implemented CDSSs, as it is more difficult to introduce a system among a larger sample of the target group. We assume that participants in our sample were somewhat self-selected with respect to attitude to and experience with working with ICT systems. However, even among these early adopters the mere dissemination of an innovation did not seem to be sufficient, emphasizing the importance of complementing dissemination with other strategies [36]. A proper introduction of the CDSS among its target users therefore seems a first important step to improve CDSS usage.

Within the group of barriers related to the evaluation of the features of the CDSS itself, we found that a high intensity of alerts, irrelevant alerts and a lack of flexibility and learning capacity of the CDSS were commonly reported. These barriers are all related to a perceived mismatch of the content of the CDSS with the varying needs of different users or user groups. They may particularly apply in a primary care setting in which different types of PCPs work with the same CDSS covering multiple disease areas of care and multiple types of alerts. Information needs [37] and therefore the perceived usefulness or relevance of alerts vary greatly across professions and even within one group of healthcare providers [4]. Improving the flexibility and learning capacity of CDSSs and thereby increasing options to tailor the decision support to the varying needs of users seems therefore particularly important.

A perceived too high intensity or irrelevant alerts may result in ‘alert fatigue’ and thereby ignoring of alerts, a phenomenon which has been discussed before [9, 38]. Irrelevant alerts, alerts that are not serious or that are repeatedly shown are the most common reasons to override them [39] and it is estimated that as much as 96 % of the alerts are overridden [39–41]. One way to deal with this alert fatigue is to demand reasons from clinicians before they can over-ride or by pass the reminders [9]. Using these so called ‘highly-insistent alerts’, however, can also be frustrating to clinicians. PCPs in our study rather felt that increased user personalization options are needed to ensure that the advice presented is relevant and useful at that time and thus may prevent alert fatigue. Even though the personalization functions of NHGDoc are quite unique, users still felt that these could be increased, for example by being able to set the threshold for alerts themselves. In addition, adaptive systems that learn from their PCPs’ use of the system and adapt the content accordingly, could be helpful.

Consistent with other studies we found that external barriers were also important barriers [4, 17–21]. PCPs indicated that they often do not feel comfortable spending much time on the computer during consultation. To deal with this patient-related barrier, it may be helpful to adapt the decision support to the needs of patients and to involve patients in using the decision support system during consultation. A recent review [9] showed that systems that involve both practitioner and patient are at least more effective in terms of improving quality of care. Another way to deal with this barrier, that also partly addresses the perceived too limited time and extra work it requires to work with the CDSS, is to rearrange patient consultation. In the Dutch setting, as in most other settings, in principal there is no time scheduled for working with CDSSs. More preparation time before actual patient consultation, could be helpful in optimally using the CDSS, while at the same time focusing more at the patient during actual consultation. Whether one of these approaches is feasible and effective needs to be further explored.

Our study has several limitations. First, this focus group study represents the opinions of only a small sample of the target group, questioning the generalizability of our findings [42]. However, our sample corresponds quite well in terms of basic characteristics to the total population of PCPs. Where older GPs were initially somewhat overrepresented in our study, we complemented our sample by adding some GP trainees to the focus groups. Therefore, we assume to have described substantial variation in perceived barriers to using CDSSs in primary care. Moreover, the aim of this study was to identify all possible barriers qualitatively rather than quantifying their relative importance. The framework of barriers to using CDSSs that emerged can be used as input for quantitative studies assessing the relative importance of the barriers.

Results of our study can be useful in designing or adapting CDSSs that are tailored to the identified barriers of their specific user groups in primary care and thus are potentially more effective in improving quality of care than CDSSs using non-user-centered designs [43–45]. However, we explored perceived barriers rather than actual barriers, which follow from PCPs’ perceptions of the situation. These may not accurately reflect the (whole range of) barriers. Whether they are actual or perceived may directly affect the effectiveness of interventions to address these barriers. Complementing this type of research with more objective types of research seems therefore useful. Future trials should directly compare effects of characteristics of CDSSs [9], such as the format and lay-out of a CDSS or certain functionalities of CDSSs.

## Conclusions

Multiple-domain covering CDSSs implemented at a large scale elicit a diverse range of barriers among the target group, of which some of them may be more prominent compared to studies focusing on specialized CDSSs within small-scale academic settings. Lack of knowledge about the system is an important barrier, emphasizing the importance of a proper introduction of the system, particularly when implemented at a larger scale. Additionally, barriers related to a perceived mismatch of the content of the CDSS with the varying needs of users or user groups may be particularly relevant in a primary care setting in which CDSSs cover multiple disease areas and target multiple groups of users. Designing flexible and adaptive systems that can be used to provide decision support tailored to the needs of different users and user groups should therefore be the main target of CDSS interventions.

## Abbreviations

CDSS: Computerized decision support system
PCP: Primary care practitioner
GP: General practitioner
GP trainee: General practitioner in training
PN: Practice nurse
DCGP: Dutch College of General Practitioners (NHG in Dutch)
EHRS: Electronic health record system
ICPC codes: International Classification of Primary Care codes
NHG lab codes: Codes used by the Nederlands Huisartsen Genootschap (DCGP in English) to classify laboratory and other diagnostic tests and results
ATC codes: Anatomical Therapeutic Chemical Classification System codes
